# Not All Herbals are Benign: A Case of Hydroxycut-induced Acute Liver Injury

**DOI:** 10.7759/cureus.6870

**Published:** 2020-02-04

**Authors:** Neelam Khetpal, Bayarmaa Mandzhieva, Sonia Shahid, Akash Khetpal, Akriti G Jain

**Affiliations:** 1 Internal Medicine, Florida Hospital, Orlando, USA; 2 Internal Medicine, AdventHealth, Orlando, USA; 3 Internal Medicine, Karachi Medical and Dental College, Karachi, PAK; 4 Internal Medicine, Dow University of Health Sciences, Karachi, PAK

**Keywords:** hydroxycut, dietary supplements, liver failure, drug toxicity, weight loss

## Abstract

Dietary supplements do not need prior Food and Drug Administration (FDA) approval before they are sold to the public per Dietary Supplement Health and Education Act of 1994 (DSHEA). Reporting serious dietary supplement related adverse reactions is voluntary. Hydroxycut is a brand of dietary supplements that are marketed as a popular weight loss product that contains multiple herbal constituents. Due to its potential hepatotoxic effects, FDA issued a warning in 2009 and recommended that consumers discontinue use of Hydroxycut. Hydroxycut was recalled from the market but a reformulated herbal mix is now available again. We are presenting a case of acute liver injury associated with Hydroxycut. The prominent pattern of liver injury is severe hepatocellular injury with the striking elevation of the aminotransferase levels and minimal abnormalities in alkaline phosphatase levels. It can sometimes cause severe hepatocellular necrosis.

## Introduction

In the United States (US), use of nutritional supplements is widespread but largely unregulated [1]. Obesity has become an increasingly important public health problem in the United States. Recent data show that more than 30% of adults are obese and 65% overweight [2].

Hydroxycut is a herbal weight loss supplement that has been linked to liver injury. Over the past decade, the components of Hydroxycut have been modified, yet new cases of liver injury have continued to emerge [3]. Consumption of dietary supplements in US has doubled to 18.9% of adults admitting their use only between 1999 and 2004 [4,5]. Some investigations report their consumption up to 47% in certain subgroups such as elderly and non-smoking women with higher education [6].

The rising popularity of dietary supplements is probably because of an increased awareness of consumers towards health in general and the desire to prevent diseases by an optimized nutritional status, and the persuasion that these treatments are safe [5].

Health advisories are issued regarding these products, but these may not be heeded by the general public as they are unaware of the consequences of taking an unregulated substance promoted for its health benefits [7]. At present, several products labelled as Hydroxycut are available and still widely used.

## Case presentation

A 22-year-old obese female presented to the emergency department with complaints of chest pain, progressive fatigue, palpitations and shortness of breath for two days. She also noticed tremors in her hands. She denied any abdominal pain, nausea, vomiting, fever, chills or rash. She reported taking a herbal supplement for weight loss, named Proclinical Hydroxycut, two capsules daily for about three months. She denied alcohol use, recent travel, other medications/supplements, family history of autoimmune diseases.

On admission, vitals were significant for tachycardia with heart rate 113 beats per minute and oxygen saturation was 84% on room air. Physical examination was remarkable for obesity with body mass index (BMI) 41, mild asterixis bilaterally. No encephalopathy, scleral icterus or hepatosplenomegaly was found. Laboratory analysis showed leukocytosis with white blood cell count of 24 x 10^3^/ul (4.4-10.5 103/ul), severe transaminitis with alanine aminotransferase (ALT) 2399 U/L (4-51 U/L), aspartate aminotransferase (AST) 4040 U/L (5-46 U/L),alkaline phosphatase level 72 U/L (40-129 U/L), total bilirubin 0.6 mg/dl (0.1-1.5 mg/dl), International Normalized Ratio (INR) 1.4 (0.8-1.2).

**Figure 1:**
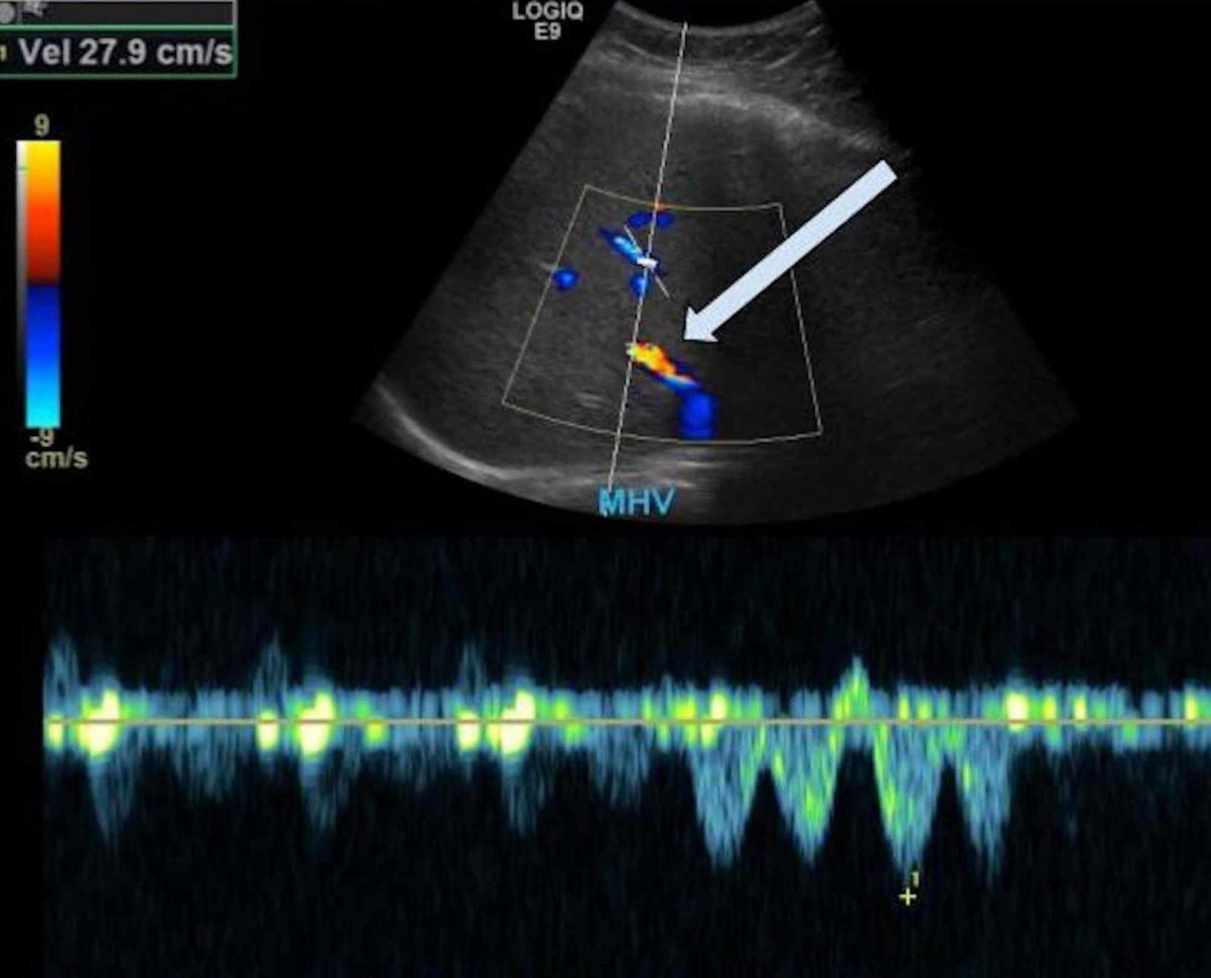
Ultrasound abdominal Doppler (liver portal-venous system) Hepatomegaly and normal Doppler ultrasound examination of the hepatic vessels. Middle hepatic vein (MHV) velocity (Vel) is 27.9 cm/s (arrow). Patent with normal waveform.

**Figure 2:**
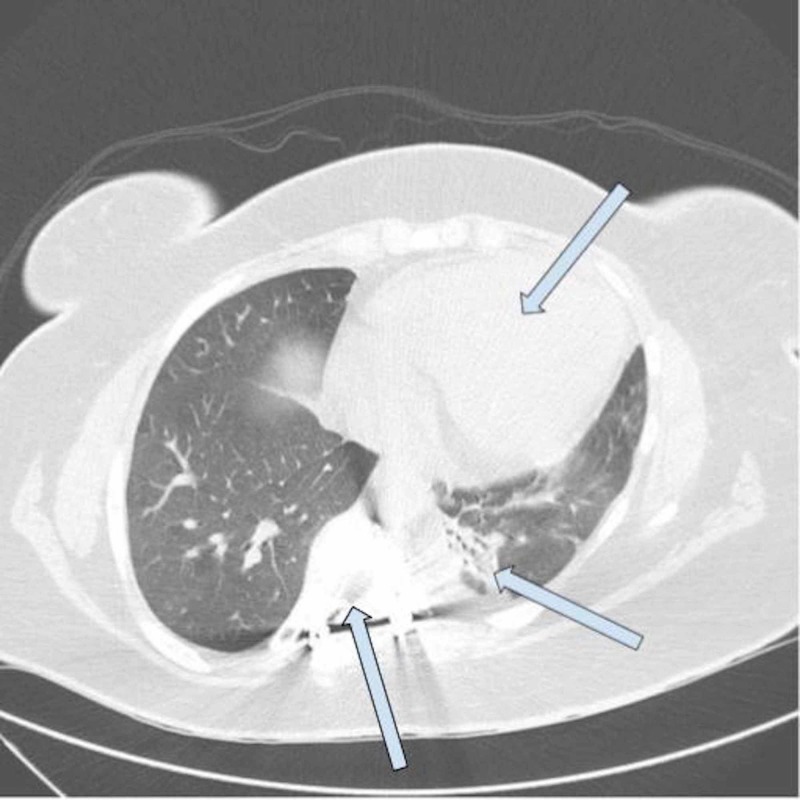
Computed tomography (CT) chest without contrast Scoliosis within the thoracic spine. Minimal atelectasis of left lower lobe. No pleural effusions. Moderate cardiomegaly with no pericardial effusion.

Investigations for other etiologies of acute liver injury including hepatitis panel, Tylenol level, antinuclear antibody, anti-smooth muscle antibody, anti-mitochondrial antibody, ceruloplasmin level, ferritin level, and serologies for Ebstein-Bar virus (EBV), herpes simplex virus (HSV) and cytomegalovirus (CMV) were unremarkable. Abdomen Ultrasound showed hepatomegaly and doppler studies of the liver were unremarkable (Figure 1). Computed tomography (CT) scan of the chest showed developmental abnormalities like scoliosis and lung hypoplasia (Figure 2).

**Figure 3:**
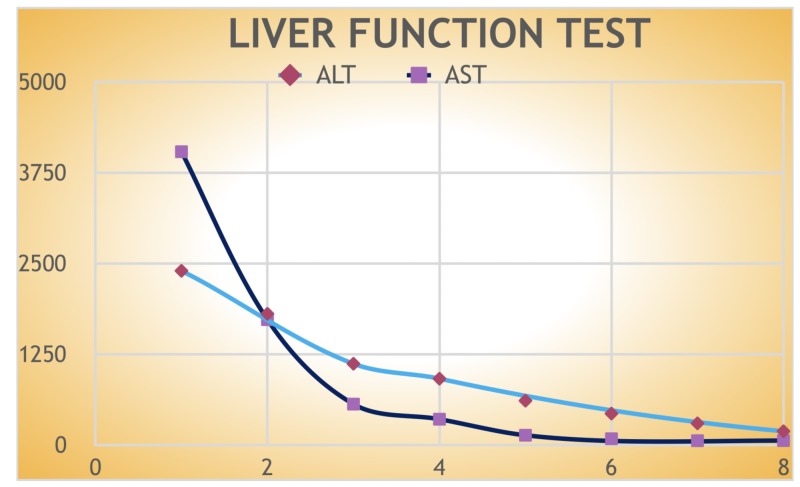
Graphical representation of transaminases trend from the day of admission until the day of discharge

She was diagnosed with acute drug-induced liver injury likely due to Hydroxycut and treated with N-acetylcysteine. Transaminases trended down during the course of hospitalization after Hydroxycut was stopped and levels were ALT 189 and AST 61 on day 8 (Figure 3). She was discharged in stable condition to follow up outpatient with hepatology. 

## Discussion

Drugs are a significant cause of liver injury. More than 900 drugs, toxins, and herbs have been reported to cause liver injury, and drugs account for 20%-40% of all cases of fulminant hepatic failure [7].

Hydroxycut is the commercial name for multi-ingredient nutritional supplements, marketed for weight loss, fat burning and body building. Hydroxycut products containing Ephedra were recalled from the market in 2004 due to cardiotoxicity associated with it. In 2009, approximately 23 cases of hepatotoxicity were associated with Hydroxycut formulations and Food and Drug Administration (FDA) issued a warning against the use of Hydroxycut products and recalled it from the market [8]. Several new formulations of Hydroxycut are still available in the market as popular weight loss products. 

Table 1 below lists several of the products with their full names and ingredients as listed on the product labels [8]. 

**Table 1:** Selected Hydroxycut products (April 2018)

Product Name	Condition	Major Listed Ingredients
Hydroxycut Hard Core Elite [Muscle Tech]	Weight loss, fat burning, enhanced energy and mental focus	Caffeine [270 mg], L-threanine [100 mg], Yohimbe extract [56.3 mg], Coleus forskohlii extract [100 mg], Green coffee extract [Coffea canephora robusta seed: 200 mg], Cocoa extract [100 mg: supplying theobromine], Yohimbe extract [56.3 mg]
Hydroxycut Hardcore CLA Elite [Muscle Tech]	Weight loss, fat burning, enhanced energy and mental focus	Conjugated linoleic acid [CLA: 1000 mg], L-carnitine [250 mg], Garcinia indica extract [250 mg], Robusta coffee bean extract [200 mg], Raspberry ketone [125 mg}
Pro Clinical Hydroxycut Lose Weight	Weight loss	Calcium (145 mg), Robusta coffee bean extract (C. canephora robusta), Papaya, Blackberry, Saffron extract, Caffeine (200 mg), Maqui (Aristotella chilensis), Amia extract (Phyllanthus)
Pro Clinical Hydroxycut Gummies	Weight loss	Thiamine (1.5 mg), Riboflavin (1.7 mg), Vitamins B6 (1 mg) and B12 (1.2 mcg), Folic acid (400 mcg), Pantothenic acid (10 mg), Robusta coffee extract (200 mg).
Pro Clinical Hydroxycut Caffeine Free	Weight loss	Calcium [150 mg], Robusta coffee extract, papaya, maqui, blackberry, amla extract, saffron extract
Pro Clinical Hydroxycut Instant Drink Mix	Weight loss	Hydroxycut Blend [340 mg] with Robusta coffee extract, papaya, blackberry and saffron extract; and HydroxyBoost with caffeine [135 mg], Maqui and Amla extract
Hydroxycut Max for Women	Weight loss	Folic acid (200 mcg), Biotin (300 mcg), Iron (2 mg) Caffeine [225 mg], Mango, Kiwi, Avocado oil, Robusta coffee extract, hydrolyzed collagen, silicon dioxide
Hydroxycut Platinum	Weight loss	Green coffee bean extract [200 mg], Red mango extract, white kidney bean extract, Ashwagandha extract, Bacillus coagulans, Caffeine [200 mg], Choline, L-theanine, Huperzine-A, Cherry stem, Lemon and Tangerine concentrates, Vitamins A, B6, B12, C, D, E and K, Folic acid, Riboflavin, Niacin, Biotin, Iron, Iodine, Pantothenic acid, Zinc, Selenium Copper and Chromium
Hydroxycut Black	Weight loss	Caffeine (200 mg), Robusta coffee bean extract (C. canephora robusta: 200 mg), Alpha lipoic acid (150 mg), Yohimbe extract, Black caraway extract, Purslane extract, Arugula extract, Chicory extract
Hydroxycut Max!	Weight loss	Folic acid (200 mcg), Biotin (300 mg), Iron (2 mg), Caffeine (225 mg), Mango, Kiwi, Avocado oil, Robusta coffee extract, hydrolyzed collagen

The specific ingredient of Hydroxycut responsible for acute liver injury has not been identified. Of various components, chromium, Garcinia cambogia extract and Camellia sinensis of green tea extracts have been mostly implicated as the components associated with hepatotoxicity [7].

Hydroxycitric acid (HCA) is the main component of Garcinia cambogia which is believed to be the main culprit for its toxicity. HCA is an inhibitor of the adenosine triphosphate (ATP) citrate lyase so it blocks de novo synthesis of fatty acids and helps in weight reduction. Recently, a fatal case of liver failure was reported in a patient taking montelukast and HCA, believed to be caused by the synergistic effect of two drugs.

Camellia sinensis is the scientific name of green tea, which is thought to be very safe by the general public for weight loss, anti-cancer, and anti-oxidant properties. Green tea contains catechins - major antioxidants. The most active catechin is epigallocatechin 3-gallate (EGCG), which is believed to be responsible for the antioxidant activity of green tea extract but also causes acute hepatocellular injury in high doses especially in combination with other environmental factors [7-9].

The onset of Hydroxycut-induced liver injury is usually within 2-12 weeks. The most common presenting features are usually nausea, abdominal pain, fatigue, and jaundice. The dominant pattern of injury is usually hepatocellular with marked elevation of transaminases. Mortality has been reported to be 10% overall in cases presenting with jaundice. Most of the non-fatal cases of Hydroxycut-induced liver injury are self-limited and resolve within 1-3 months but few cases can be fatal and associated with grave outcomes [8]. 

Due to the variability of presentation and lack of specific diagnostic markers, causality to a specific product is difficult to determine in cases of drug/herbal-induced liver injury. Drug-induced liver injury is usually a diagnosis of exclusion. A high index of suspicion is needed and requires a thorough history taking and physical examination. It is also important to read the product label for various ingredients as some of them may have a ‘signature’ side effect previously reported in the literature. The temporal relationship between drug intake and onset of injury (challenge) and resolution of injury and drug cessation (dechallenge) strengthens the causality of drug-induced liver injury. It is further enhanced by recurrence of symptoms with preexposure to the same agent (rechallenge) [8,10,11].

Several scoring systems have been proposed to aid with causality in drug-induced liver injury such as Naranjo Probability Scale, Roussel Uclaf Causality Assessment Method (RUCAM) and its modification known as the Maria and Victorino (M & V) System. Naranjo Scale is not specific for liver injury and can be used for any drug-associated organ injury whereas RUCAM and M&V are specific for drug-induced liver injury

RUCAM scale calculated for our patient was 9, which is associated with a high possibility of drug-induced liver injury (RUCAM Causality Scoring: 0 or <= excluded, 1-2= unlikely; 3-5= possible; 6-8 probable; >8 highly possible) [8].

Despite multiple reports of side effects associated with weight loss and other over the counter herbal products, dietary supplements are not subjected to the same regulations by FDA as licensed drugs and FDA is not authorized to review dietary supplements for safety and efficacy prior to marketing. MedWatch is an FDA’s voluntary adverse event reporting program which is used to share critical information to the general public and medical community. It also disseminates public safety alerts regarding the adverse effects of dietary supplements [12].

## Conclusions

Our case emphasizes the importance of minimizing the use of over the counter dietary supplements which can lead to catastrophic side effects. Oversight of their use by clinicians remains poor and detailed history taking should be obtained. Further studies are needed to better clarify the mechanisms and patterns of injury. This case also demonstrates the need for better post-marketing surveillance of herbal and dietary supplements and more stringent regulation by the FDA as is in place for prescription drugs.
